# Effect of old age on the subpopulations of enteric glial cells in human descending colon

**DOI:** 10.1002/glia.24272

**Published:** 2022-09-20

**Authors:** Nicholas Baidoo, Gareth J. Sanger, Abi Belai

**Affiliations:** ^1^ School of Life and Health Sciences University of Roehampton London UK; ^2^ Blizard Institute, Faculty of Medicine and Dentistry Queen Mary University of London London UK

**Keywords:** aging, circular muscle, enteric glial cells, GFAP, human colon, myenteric plexus, S‐100, Sox‐10, *Taenia coli*

## Abstract

Old age is associated with a higher incidence of lower bowel conditions such as constipation. Recent evidence suggest that colonic motility may be influenced by enteric glial cells (EGCs). Little is known about the effect of aging on the subpopulation of EGCs in the human colon. We assessed and compared the pattern of distribution of EGCs in adult and elderly human colon. Human descending colon were obtained from 23 cancer patients comprising of adults (23–63 years; 6 male, 7 female) and elderly (66–81 year; 6 male, 4 female). Specimens were serially‐sectioned and immunolabeled with anti‐Sox‐10, anti‐S100 and anti‐GFAP for morphometric analysis. Standardized procedures were utilized to ensure unbiased counting and densitometric evaluation of EGCs. The number of Sox‐10 immunoreactive (IR) EGCs were unaltered with age in both the myenteric plexus (MP) (respectively, in adult and elderly patients, 1939 ± 82 and 1760 ± 44/mm length; *p* > .05) and submucosal plexus; there were no apparent differences between adult males and females. The density of S100‐IR EGCs declined among the elderly in the circular muscle and within the MP per ganglionic area. In the adult colon, there were more S100‐IR EGCs distributed in the circular muscle per unit area than the *Taenia coli.* There was little or no GFAP‐IR EGCs in both adult and elderly colon. We concluded that aging of the human descending colon does not result in a loss of Sox‐10‐IR EGCs in the MP and SMP but reduces S100‐IR EGCs density within the musculature. This alteration in myenteric EGCs density with age may contribute to colonic dysfunction.

## Main Points

- The number of Sox‐10 immunoreactive (IR) enteric glial cells (EGCs) were unaltered with age in both the myenteric and submucous plexus.
- The density of S100‐IR EGCs declined among the elderly in the circular muscle and within the myenteric plexus per ganglionic area.
- There were little or no GFAP‐IR EGCs in both adult and elderly colon.

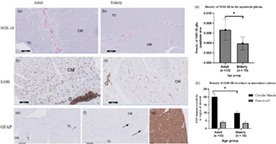

## 1 INTRODUCTION

In humans, there is an increased incidence of conditions such as fecal impaction, constipation and incontinence among the elderly population (Bharucha & Lacy, 2020, see Gau et al., 2022), often reducing the quality of life and independence (Wald et al., 2007). Changes in lifestyle (e.g., diet, level of exercise; Liu et al., 1993) and intake of one or more prescribed medications (e.g., NSAIDs, calcium‐channel antagonists and calcium supplements; Wawruch et al., 2012) can disrupt gastrointestinal (GI) tract motility in the elderly. However, physiological and pharmacological studies in humans (Broad et al., 2019; Cibert‐Goton et al., 2020; Madsen & Graff, 2004; Zizzo et al., 2022) also suggest a decline in neuromuscular and afferent nerve functions in the aging colon, associated at least in part with “inflammageing” (Mauro et al., 2022) and increased expression of post‐mitotic cellular senescence‐like activity within enteric neurons (Palmer et al., 2021).

The enteric nervous system (ENS) plays an important role in the control of colonic motility, consisting primarily of neurons and enteric glial cells (EGCs) (Furness et al., 2014). Studies on the effects of aging on human intestinal functions have mostly focussed on the muscle, enteric and extrinsic neurons (see above), but little is known about the presence or functions of EGCs in advancing age. EGCs are in the myenteric plexus (MP) and submucosal plexus (SMP) as well as the muscularis externa of the GI tract (Furness et al., 2014). In addition to the active roles of EGCs in controlling different aspects of GI function (see Seguella & Gulbransen, 2021), these cells surround neuronal cell bodies and processes, providing functional EGCs‐neuron communication (see Pawolski & Schmidt, 2020). Furthermore, it has been reported that EGCs provide immunological support (Chow & Gulbransen, 2017), exhibit sufficient plasticity to form new neurons or replace dying neurons (Laranjeira et al., 2011) and help to regulate gut motility (McClain et al., 2014; McClain et al., 2015).

In rats, significant reductions in S100 immunoreactive (IR) EGC density per ganglion (within the MP) occurred in the duodenum, jejenum and colon sampled from aged (26 months of age) animals compared to the younger group (5–6 months of age) (Phillips et al., 2004); the authors inferred that EGCs loss was proportional to neuronal death, suggesting interdependency between the two cell types. In another study with mouse colon (2 months vs 12 months old), altered motility with increasing age was associated with reduced EGCs expression of connexin‐43 mRNA and protein within the MP (McClain et al., 2014). However, these data from animal studies although valuable, cannot necessarily be extrapolated to humans as EGC populations differ between species, gender and region of the gut wall with respect to their morphometric and functional characteristics (Boesmans et al., 2015; Costagliola et al., 2009; Hoff et al., 2008).

There has been one study that looked at the effect of aging on the numbers of EGCs in human colon. In this study carried out on proximal and distal colon, an apparent age‐related decline in the numbers of S100‐IR EGCs within human myenteric ganglia (isolated by laser microdissection from adult (48–58 years) and elderly (70–95 years) patients) was not statistically significant (Hetz et al., 2014). In other studies, S100‐IR EGCs in human descending colon were found to be decreased in both the MP and SMP from patients with slow transit constipation (Bassotti et al., 2006).

To date, there is no single pan‐EGC marker capable of identifying all populations of EGCs in the GI tract. The most recently accepted marker capable of labeling most (but not all) EGC populations is the sex‐determining gene SRY on the Y‐chromosome‐related HMG‐box (Sox) 10, one of the earliest neural crest cell markers of ENS progenitors (Paratore et al., 2001). In addition, EGCs are known to express the calcium‐binding protein S100 (Ferri et al., 1982) and the intermediate filament glial fibrillary acidic protein (GFAP; Jessen & Mirsky, 1980). Further, marker expression analysis in the GI tract of adult mice showed that the majority of EGCs co‐express GFAP, S100 and Sox‐10 in the myenteric ganglia but did not reproduce the same result outside the plexus (Boesmans et al., 2015), suggesting EGCs may exhibit phenotypic variation within the intestinal wall. Other studies have shown variability in the expression of Sox10, S100, and GFAP within the ENS of adult human colon (see Grundmann et al., 2019). Nevertheless, recent findings indicate that Sox‐10 is the closest pan‐EGCs biomarker that identifies most of the glial cells, now widely utilized for EGCs quantification (Grundmann et al., 2019).

To begin to understand the effect of age on EGCs, it is vital to evaluate the presence of different functional EGC subpopulations within the sublayers of colonic wall. Presently, the main EGC populations that are recognized are; intraganglionic (Type 1: resides within the ganglion in the MP and SMP) and extraganglionic resides in interganglionic fiber tracts connecting myenteric ganglia in the plexus (Type II), those located in the mucosal region (Type III) and intramuscular layer (Type IV); these were identified based on morphology and location (Boesmans et al., 2015; Hanani & Reichenbach, 1994). We therefore investigated the effects of age on the numbers of EGCs (anti‐Sox‐10) within the myenteric and submucous plexus, and in addition, used anti‐S100 to examine the network density in the musculature and undertook a morphological evaluation with anti‐GFAP, each in adult (<65 years) and elderly (≥65 years) human descending colon.

## 2 MATERIALS AND METHODS

### 2.1 Subject selection

A total of 23 macroscopically normal descending colon tissues were obtained as surgical surplus tissues from patients undergoing elective surgery for non‐obstructing bowel cancer, following informed written consent. None of the patients had previous chemoradiotherapy or diagnosis of active inflammatory colonic disease. The sections of colon were obtained at least 5–10 cm away from the tumor and were prospectively collected until 23 patients were received; adults (23–63 years; 6 male, 7 female) and elderly (66–81 years; 6 male, 4 female). Patient records were examined for current medication and comorbidity (See supplementary sheet 1). None of the patients used in this study were diagnosed with slow transit constipation, known to affect EGCs density in human colon (Bassotti et al., 2006). This study was approved by the University of Roehampton (LSC 21/339) and by the East London (REC 10/H0703/71) Ethics Committees.

### 2.2 Procedures

Human colonic tissues were fixed, processed, embedded transversally and systematically serially sectioned as described in similar study using formalin fixed, paraffin embedded tissues (Ippolito et al., 2009). To blind the investigators during analysis to the age and sex of the patients, slides were assigned codes during microtomy. Before immunohistochemistry staining was performed, sections were deparaffinized, rehydrated and stained for routine hematoxylin and eosin (H&E). None of the colon sections, based on H&E assessment, had active inflammation, tumor, and structural abnormalities (Feakins & British Society of Gastroenterology, 2013).

#### 2.2.1 Immunohistochemistry

A minimum of eight sections at 16 μm separation per sample were loaded on an automated Ventana BenchMark XT system (Roche, Ventana Medical Systems Inc., Tucson) and pre‐treatment carried out in mild cell conditioning solution (CC1) (Roche, Ventana Medical Systems, Inc., Tucson) for anti‐GFAP (1:100; Monoclonal rabbit; Cellmarque, CA), S100 antibodies (1:1000; Polyclonal rabbit; Dako, Santa Clara, CA) and anti‐Sox‐10 (1:100; Polyclonal rabbit; Cellmarque, CA). EGC immunostaining visualization was based on horseradish peroxidase or alkaline phosphatase linked detection system, following the manufacturer's recommendations. Positive and negative controls were performed in colon (anti‐S100 and Sox‐10) and anonymous brain control section (anti‐GFAP), with or without primary antibodies. Non‐specific structures were counterstained with Harris hematoxylin for 5 s. Sections were then dehydrated in graded series of alcohol, cleared in xylene, mounted with Pertex and coverslipped with glass slide (Sakura, Tokyo‐Japan). IR‐EGC structures stained red (anti‐Sox‐10), or brown (anti‐S100 and anti‐GFAP) and non‐specific background was blue.

#### 2.2.2 Evaluation of EGC numbers within the MP and SMP

**FIGURE 1:**
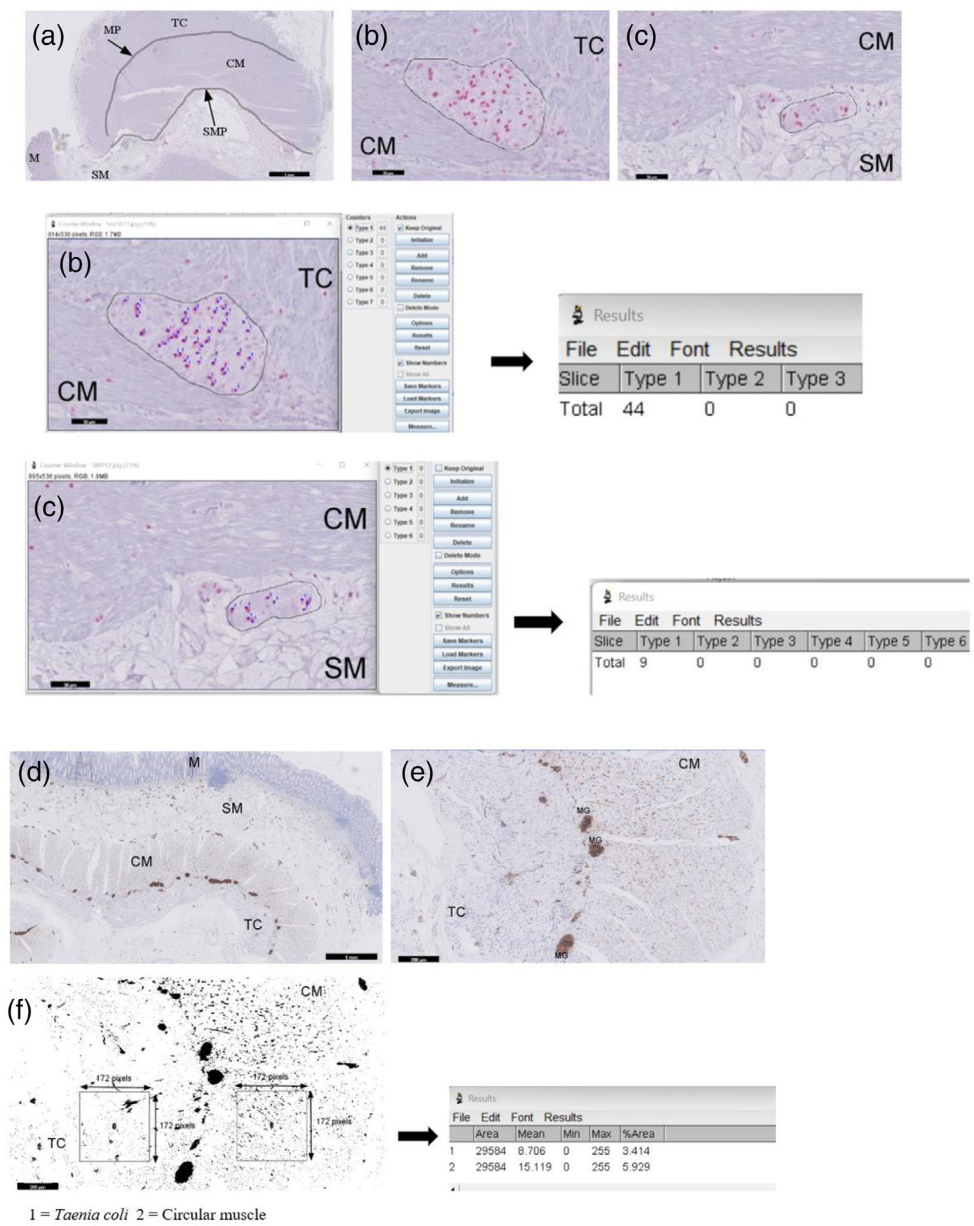
Methodology used to count number of Sox‐10‐immunoreactive (IR) enteric glial cells (EGCs) and evaluate the density of S100‐IR EGCs in human colon. Panel (a) is a representative of a full thickness Sox‐10‐stained adult colonic tissue section marked with a trace tool applied with image J processing on myenteric plexus (MP) and submucous plexus (SMP) to determine length. Scale bar 1 mm. Respectively, panel (b and c) shows myenteric and submucous ganglia, marked with a freehand tool to count the number of Sox‐10‐ IR enteric glia cell bodies. Each Sox‐10‐IR cell bodies were counted with a cell counter plugins and results of number of EGCs in the ganglion presented. Scale bar 50 μm. Panel (d) shows a full thickness of formalin‐fixed paraffin embedded tissue section labeled with an antibody to S100 to identify enteric glia structures. Scale bar 1 mm. Panel (e) represents a section of the muscularis externa imported to image J to determine the density of S100‐IR enteric glia structures in the circular muscle (CM) and *Taenia coli* (TC). The density of S100‐IR is the amount of positive pixel per region of interest. Scale bar 200 μm. Myenteric ganglion (MG). In panel (f), the image was threshold and binarized so that only S100‐IR structures were included. A sample area box of 172 × 172 pixels was drawn to measure the positive pixels of S‐100‐IR structures in the *Taenia coli* and circular muscle. An area of 1 mm^2^ for each TC and circular muscle was quantified per patient. The amount of S‐100‐IR in the *Taenia coli* (1) and circular muscle (2) was automatically calculated and presented in results. Scale bar 200 μm

All immuno‐stained sections were scanned (Phillips IntelliSite ultra‐Fast Scanner, NOCTN442, Netherlands) and digitally viewed. Sox‐10 antibodies labels only the EGCs cell bodies (Bondurand & Sham, 2013). Counting of EGC bodies with anti‐Sox‐10 were performed via digital visualization in a blinded fashion to patient age. Digital scanning of the stained slides prior to analysis ensured that all tissue sections were systematically evaluated, avoiding the possibility that areas of few or many Sox‐IR EGCs in the MP and SMP were inadvertently preferentially analyzed. For quantification of Sox‐10‐IR EGCs in both the MP and SMP, we applied a trace tool in the viewing software programme (Philips Digital Pathology Viewer, Netherlands) to draw a line (in mm) along the contours for both plexuses (Figure 1a). Within that length (in mm), all Sox‐10‐IR EGCs and ganglia in the MP and SMP were counted (Figure 1b,c) with Cell counter in ImageJ (Version 1.53f51; Schneider et al., 2012). At least a minimum of 4 mm and a maximum of 5 mm length of the MP and SMP were measured per section/patient. For each EGCs analysis, a median of 10 sections per patient were used. The number of ganglia, the number containing Sox‐10 IR and the number of Sox‐10 IR cells within each ganglia and in the length of both MP and the SMP plexus were counted and expressed as number of EGCs/ganglion and number of EGCs/mm length of the plexus. The definition of MP was an area found between the circular muscle (CM) and the *Taenia coli* (TC). All Sox‐10 IR EGCs bodies located within the ganglion (intraganglionic/mm length) of the plexus was first evaluated for both MP and SMP. We then assessed Sox‐10 IR EGCs bodies outside the ganglion but along the contour of the MP (extraganglionic/mm length). Total number of EGCs in the MP is the summation of intraganglionic and extraganglionic Sox‐10 IR EGCs. For SMP, count of Sox‐10 IR EGCs number was based on intraganglionic/mm length of plexus. Due to the poor arrangement of ganglion within the SMP, extraganglionic Sox‐10 IR EGCs were not included in the count. The SMP was in a region toward the edge of the circular muscle (CM) layer containing a well‐defined encapsulated structures with at least two Sox‐10‐IR EGCs bodies. Quantitative estimation of EGCs counts in the ganglion using formalin‐fixed paraffin‐embedded tissue section was adapted from a slightly modified method (Ippolito et al., 2009; Swaminathan & Kapur, 2010). (1) EGCs must be within the ganglion. (2) All visible distinct red nuclear stain was counted. (3) An overlapping or continuous region of red staining in the presence of two distinct nuclei was counted as two cell bodies. (4) Extraganglionic Sox‐10‐IR EGC bodies must be within the contour of the MP.

#### 2.2.3 Densitometric assessment of intramuscular EGC network

Sections were labeled using anti‐S100 marker capable of identifying both EGC processes and cell bodies to evaluate changes in S100‐IR EGC density in the myenteric ganglia and the muscularis externa. Immunolabeled sections were scanned and digitally visualized as described above. For each stained section, the entire CM and TC were individually circumscribed with a tracing tool to capture an area of 1 mm^2^ for each region in viewing software. Images were then imported to ImageJ (Version 1.53f51) for processing (Figure 1d,e). At least eight sections per patient were used for analysis and the values of results per patient was averaged. Respectively, a minimum area of 8 and 4 mm^2^ at 4 μm tissue thickness of CM and TC were evaluated. For analysis of S100‐IR staining per region of interest, images were thresholded to remove interfering background and binarized (Figure 1f). The density of S100‐ IR EGCs staining within the area measured, was determined from the positive pixel labeling per region of interest in the musculature and in myenteric ganglion per unit area.

#### 2.2.4 Morphological assessment of the EGCs

GFAP marker labels EGCs processes and has been routinely used for tracking changes in EGCs morphology within the gut compartment (see Grundmann et al., 2019). For qualitative evaluation of GFAP‐IR EGCs, stained slides were scanned and digitally viewed as described above. Morphological descriptions of the absence/presence of GFAP‐IR EGCs processes within the colonic sublayers were evaluated. In this study, sections with artifacts and those with irregular staining patterns were excluded from the analysis. However, where an artifact is seen for example in the CM, the unaffected TC of that section was still analyzed and included in the study. A mean of 23 mm of MP and SMP/Sox‐10 EGC bodies per patient was evaluated for EGCs count.

### 2.3 Statistical analysis

The Kolmogorov–Smirnov test for normality was applied on all values for EGCs density and count which demonstrated that data were normally distributed for each group investigated. Differences in EGCs evaluation between the adult and the elderly groups were compared by two‐tailed independent student's test and in multiple comparison between different colonic sublayers, a one‐way ANOVA with Bonferroni post‐hoc was performed using the Statistical Package for Social Science (IBM Corp. Released 2019. IBM SPSS Statistics for Windows, Version 26.0. Armonk, NY) software. Power analysis based on preliminary EGCs investigation was performed prior to sample collection to determine the minimum number of subjects sufficiently able to detect any age‐related alterations for the present study. This analysis confirmed that power of 95% requires nine (9) patients from each group for immunohistochemical analysis. Due to variability of colonic tissue size and arrangement of myenteric and submucous ganglia within and between groups, all data were considered and expressed as a mean (±Standard error of mean). GraphPad prism software (Avenida de la Playa La Jolla, USA) was used to plot graphs. *p* ≤ .05 were chosen for statistical significance. Unless otherwise specified, *n* represent the number of patients.

## 3 RESULTS

### 3.1 Immunolabeling of EGCs with Sox‐10, S100 and GFAP in adult human colon

**FIGURE 2:**
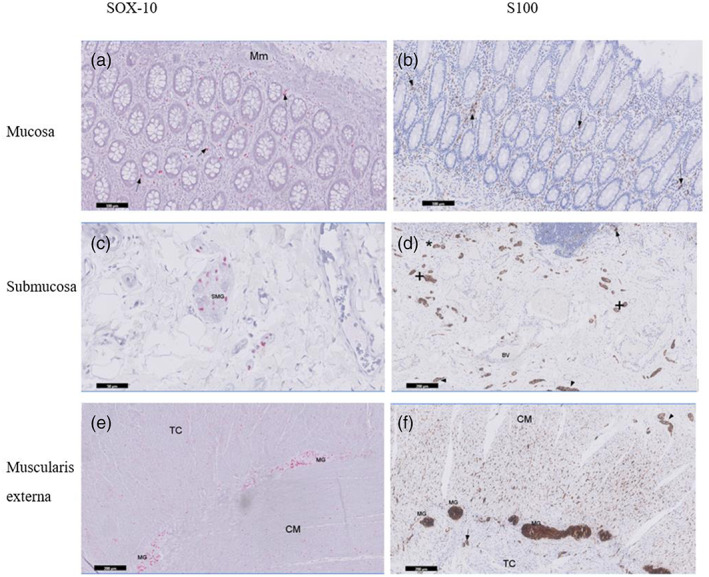
Expression of enteric glial cells (EGCs) markers with Sox‐10 (red‐stained) and S100 (brown‐stained) antibodies in adult descending colon. Abundant Sox‐10 proteins were strictly localized to the EGC bodies whereas S100 displayed positive structures in both the glial cell bodies and processes. High expression of Sox‐10‐ and S100‐immunoreactivity structures (arrow) were morphologically detected in the mucosa (a and b), submucosa (c and d) and the muscularis externa (e and f) of adult human colon. Within the submucous layer, three different ganglia were identified. Ganglia close to the muscularis mucosae (marked by *), those found next to inner circular muscle (marked by arrowhead) and ganglia in the middle of the submucosa (marked by +). In the submucosa, enteric glia structures were seen close to the mucosal immune cells (marked by arrow) and blood vessels (BV). In the submucosa ganglia (SMG) and myenteric ganglia (MG), individual Sox‐10‐IR EGCs could be clearly identified by their intensely red stained cell bodies. EGCs structures were visibly seen within clustered in the *Taenia coli* (arrow) but this observation was absent in the circular muscle (f). CM: Circular muscle; TC: *Taenia coli*. Scale bars: A & B = 100 μm; C = 50 μm; D, E & F = 200 μm

**FIGURE 3:**
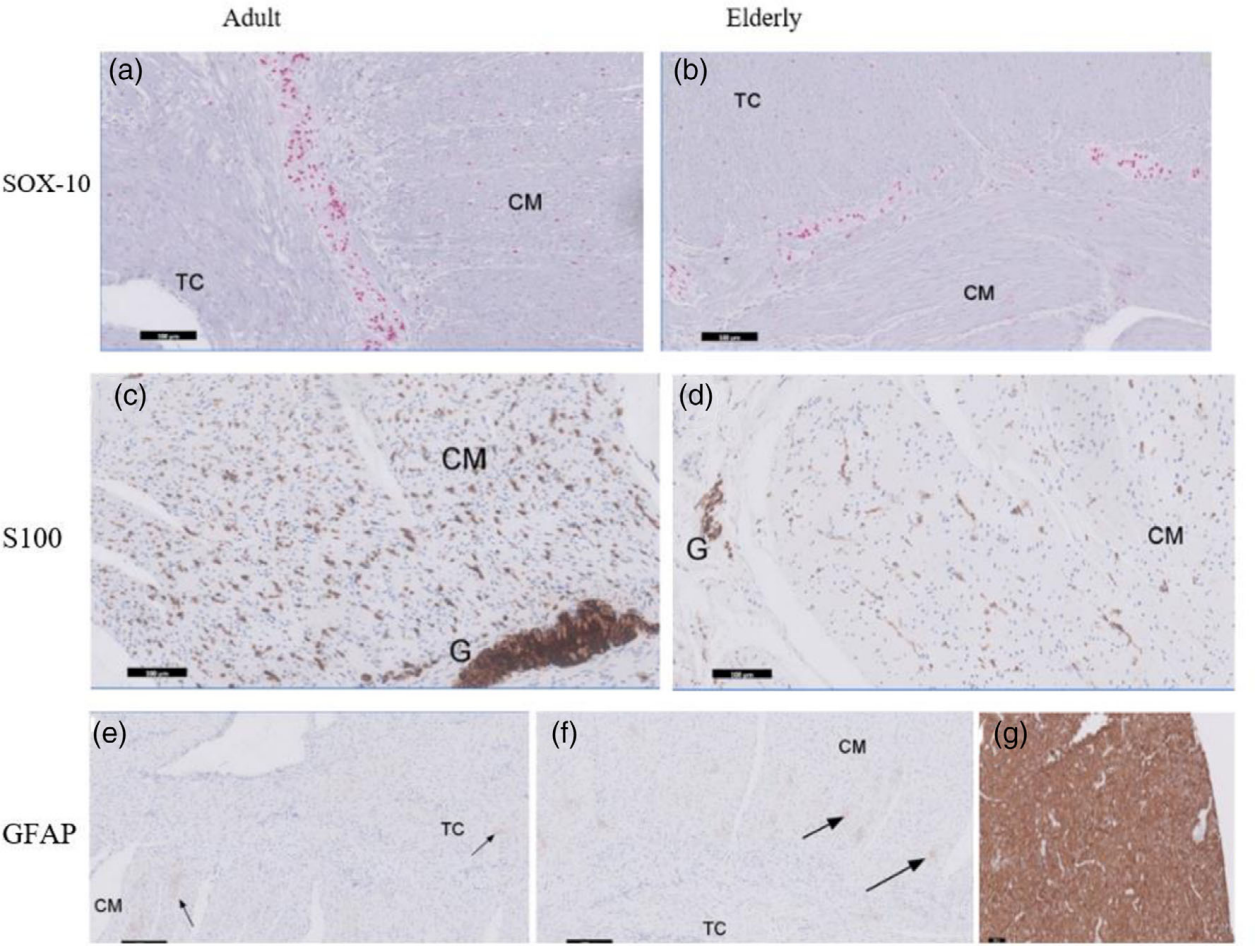
Representative examples of enteric glia staining markers expression between adult (23–63 years) and elderly (66–81 years) in human descending colon. Enteric glia cells were identified using antibodies for Sox‐10, S100 and glial fibrillary acidic protein (GFAP). Stained sections were acquired using Philips pathology scanner software and imported to ImageJ processing for analysis. In panel (a and b), data (mean ± SEM) from Sox‐10‐IR enteric glia bodies count/mm length of myenteric plexus (within ganglion and along the plexus) were considered and compared, there was no change in the number of Sox‐10 expressing EGCs between adult and the elderly (1939 ± 82 vs 1760 ± 44/mm length of plexus; *p* > .05) was observed. In panel (c and d), densitometric evaluation of S100‐IR was performed on binarized images and density of positive staining up to an area of 1 mm^2^ was measured for both circular muscle and Taenia coli in each group using ImageJ. Panel (e and f) show representative GFAP staining pattern between adult and elderly samples. GFAP‐IR EGCs were absent in all the sublayers investigated except in muscular coat layer where very low‐protein expression was observed in both the adult and the elderly samples when compared the staining intensity to positive control (g)

High levels of expression of Sox‐10 and S100‐IR structures but not GFAP‐IR, were detected in the mucosa, submucosa and the muscularis externa (Figure 2a–f). Sox‐10 proteins were localized to the EGC bodies whereas S100 stained both the EGC bodies and processes. In the MP and SMP, individual Sox‐10‐IR EGC bodies could be clearly identified by their intensely red stained cell bodies. Similarly, an appreciable brown staining of S100 EGC structures were distinguished from the background. Three different ganglia were identified within the submucosa with both Sox‐10 and S100 antibodies. Herein, ganglia is defined as neural structure containing at least two Sox‐10 IR or S100 IR structures within a defined connective tissue capsule. One (inner) was close to the *muscularis mucosae* of the mucosa layer; another was found in the middle (intermediate) of the submucosa and the third (outer) ganglia found close to inner circular muscle of the muscularis externa (see Supplementary 2 C1‐3). Microscopically, numerous intermediate submucosal ganglia predominate compared to those that lie close to the circular muscle and *muscularis mucosae* (Figure 2d). Further, there were numerous but smaller submucosa ganglia compared to those found in the MP. A few smaller structures containing S100‐IR were seen clustered within the *Taenia coli* but these were absent in the circular muscle layer (Figure 2f). Additionally, numerous nucleated and processes of S‐100‐IR EGCs were predominantly observed toward the myenteric region of the CM layer of the adult compared to the elderly samples (Figure 3c,d). The EGC marker GFAP, displayed a very weak or low expression and/or almost absent fibrillary staining pattern within the muscle layers in the adult compared to the expression levels of Sox‐10 and S100. GFAP‐IR was absent in both the myenteric and submucous ganglia as well as the mucosa layer in the adult descending colon. When all Sox‐10, S100 and GFAP labeling were qualitatively assessed within the sublayers of adult colonic wall, the result is presented in Table 1.

**TABLE 1:** Immunohistochemical expression of Sox‐10, S100 and GFAP in formalin‐fixed, paraffin embedded adult human colonic sections. Adult (23–63 years; 6 male, 7 female) samples were immunolabeled with Sox‐10, S100 and GFAP markers and were microscopically assessed by the presence (+) and absence (−) of immunoreactivity within the colonic sublayers. +/− represents very low or almost absent expression. GFAP‐IR were absent in all the sublayers of adult human colon except in muscle coat where a very low expression was sighted

	Sox‐10	S100	GFAP
Mucosa	+	+	−
Submucosa	+	+	−
Submucosa plexus	+	+	−
Circular muscle	+	+	+/−
Myenteric plexus	+	+	−
*Taenia coli*	+	+	−

### 3.2 EGC numbers in colon of the elderly

In the adult (23–63 years) samples (*n* = 13; 6 male, 7 female), an average of 1575 ± 65 and 451 ± 33 intraganglion/mm length of Sox‐10‐IR EGCs bodies were counted within the MP and SMP, respectively. The intraganglionic Sox‐10‐IR EGC numbers within the MP of the elderly (66–81 years; 6 male, 4 female) tended to be smaller (1360 ± 52 ganglion/mm length of MP) but this difference was not statistically significant (*p* = .06). Further, no age‐related changes in the number of Sox‐10 IR EGCs were noted within the SMP (451 ± 33 vs 390 ± 21/intraganglion/mm length of SMP; *p* > .05). We identified Sox‐10‐IR structures which were not within the ganglia but located adjacent and between ganglia (extraganglionic), along the trajectory of the MP. These Sox‐10 IR EGC bodies numbers were counted per millimeter length of the MP. When all data from total Sox‐10 IR EGC bodies (intra and extraganglion) count/mm of MP were considered and compared between the group, we did not detect a statistically significant change with age (1939 ± 82 vs 1760 ± 44/mm length of MP) in the MP. Similarly, the number of ganglia/mm length identified by Sox‐10 and S100 antibodies in both myenteric (2.6 ± 0.4 vs 2.2 ± 0.2) and submucous (6.2 ± 0.2 vs 6.1 ± 0.1) plexus remain unchanged. For each of these measurements, examination of patient records for the adult and elderly populations (Supplementary sheet 1) showed no clear associations with the use of medications or co‐morbidities, although the numbers were too small for a robust analysis.

### 3.3 Effect of sex on EGCs numbers

In the adult human colon, the numbers of colonic samples from males (*n* = 6) and females (*n* = 7) were too small for a robust analysis but no statistically significant sex‐related differences in the total number of Sox‐10 IR EGCs were observed within the MP (Mean EGCs: 1978 ± 32 vs 1845 ± 26/mm length of MP). Similarly, no sex related differences in the number of Sox‐10 IR EGCs were noted within the SMP (Mean EGCs: 481 ± 29 vs 421 ± 19/ganglion/mm length of SMP; *p* > .05).

### 3.4 Age‐related changes in the density of EGCs in the MP and the muscularis externa

**FIGURE 4:**
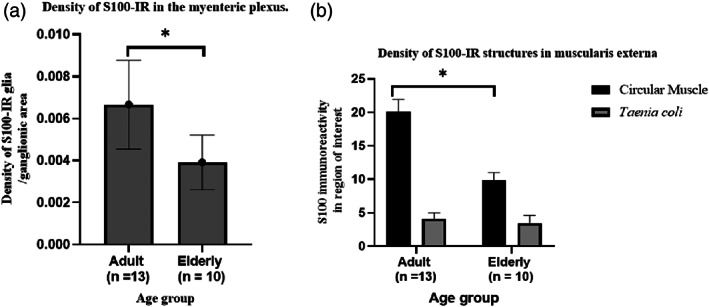
S100 immunoreactive enteric glia cell decrease with age in the myenteric plexus (MP) and circular muscle of the human descending colon. Respectively, panels (a and b) summarize age‐related changes in the density of S‐100‐IR glia per ganglion within the MP and in the muscularis externa. In both panels, stained sections were digitally scanned with Philips pathology scanner and were imported to ImageJ software for processing. Age‐related changes between adult (< 65 years) and elderly (≥ 65 years) were compared using independent t‐test. All data were expressed as means ± SEM. *p < .05

To investigate the distribution of S100‐IR structures in the muscularis externa, we first determined the density of S100 IR EGCs in the MP of the adult sample. Because of the broad staining of S100 antibodies (labeling both EGCs processes and bodies; Figure 3c,d) counting of these cells individually were not possible. We analyzed the density of S100‐IR EGCs per ganglionic area per millimeter of the MP as well as the distribution between CM and TC in adult sample. The density of S100‐IREGCs per unit ganglionic area in the MP declined with increasing age (Figure 4a). There was more S100‐IR EGCs in the adult CM than the TC (mean positive pixels: 20.1 ± 1.8 vs. 4.1 ± 0.9 per mm^2^; *p* < .05; *n* = 13). In further analysis, samples from older human colonic sections showed a statistically significant decline in S100‐IR EGCs density in the circular muscle layer compared to the adult (Figure 4b). Examination of patient records for the adult and elderly populations (Supplementary 1) showed no clear associations between these changes and the use of medications or co‐morbidities, although the numbers were too small for a robust analysis.

### 3.5 GFAP ‐IR EGCs in human descending colon

We assessed the appearance of GFAP ‐IR enteric glia cell staining marker expression between adult (23–63 years) and elderly (66–81 years) in human descending colon. GFAP ‐IR were absent in the mucosa, submucosa and in both myenteric and submucous ganglia in adult samples. Similar observations were found in the elderly samples. In the muscle coat, very low expression of GFAP ‐IR were seen at higher magnification in both age groups, however too insignificant to make a meaningful quantitation (see Figure 3e,f).

## 4 DISCUSSION

The present investigation is the first detailed analysis utilizing the three main biomarkers to provide an assessment of EGC numbers within the MP and SMP, and their density in the MP and the individual sublayers of muscularis externa in aging human descending colon. Counting of EGCs within the MP and SMP and densitometric analysis was based on a strict application of defined criteria (Ippolito et al., 2009; Swaminathan & Kapur, 2010) using formalin‐fixed paraffin‐embedded tissue sections. The principal findings indicated that aging does not affect the overall number of Sox‐10‐IR EGCs in the MP and SMP, however, decline in density of S100‐IR EGCs occurs within the MP per ganglionic area and the CM layer. In addition, this study reported that GFAP‐IR structures were absent in most of the sublayers of the colonic wall except in CM where very low expressions were identified, insufficient to give a meaningful qualitative assessment. Sex‐related differences were not found when all data were considered between adult male and female.

Sox‐10 is a useful marker as it labels the EGCs bodies and gives accurate quantification of EGCs numbers in the plexus studied. In addition, it is the only antibody that is currently known to identify the majority of the EGCs population in human gut wall (see Grundmann et al., 2019). In the MP and SMP of the elderly, Sox‐10 expressing EGCs numbers remain unaltered compared to the adult. Another finding recorded in the count of Sox‐10 IR EGCs within the myenteric ganglia alone yielded a statistically insignificant borderline, with a possible biological relevance compared to the results where both total count of Sox‐10 IR EGCs in the ganglion and those located along the trajectory of the MP were considered. The present study report a statistically insignificant overall numbers of Sox‐10 IR EGCs per ganglion/mm length of plexus in aging human descending colon. Our finding on unaltered Sox‐10 IR EGCs numbers within the submucous ganglia is similar to previously reported findings in human study (Hoff et al., 2008).

In another study, a decline in age‐related Sox‐10‐IR EGCs per ganglionic area in the MP of C57B1/6 J mice (*n* = 5–7) has been reported (Stenkamp‐Strahm et al., 2013), the authors did not account for the extraganglionic EGCs in the MP which could have altered the overall findings. The difference between the present result and the decline in Sox‐10‐IR EGCs in C57B1/6 J mice is that, apart from species specificity, the EGCs diversity is greater in humans than in rodents (Bon‐Frauches & Boesmans, 2020). In addition, the sampling method and sample size could influence the EGCs count. Our method of systematic count of EGCs within the plexuses eliminates the element of bias in random fields samplings. Further, different phenotypic EGCs are widely distributed in the sublayers of the gut wall based on morphologies and localisation. Gulbransen and Sharkey (Gulbransen & Sharkey, 2012) reported the existence of four (Type I–IV) morphological subtypes of enteric glia. The authors described Type I, II, III, and IV enteric glia localized respectively in, the ganglia, interganglionic connectives, mucosal and intramuscular layer. The very few studies investigating myEGCs number per ganglion during aging have not taken into consideration the distinct functional EGCs subtype along the interganglionic connectives which was included in the present study and this, most importantly, accounted for differences in our results on Sox‐IR EGCs numbers. Nevertheless, the present result is consistent with unaltered number of Sox‐10 expressing EGCs with age in the myenteric and submucous plexus of ileum, proximal and distal colon of Dunkin Hartley guinea pigs using whole‐mount technique (Hoff et al., 2008). A plausible explanation for the lack of change Sox‐10‐IR EGCs numbers at the MP in the elderly sample is that Sox10 stained undifferentiated neural‐crest progenitor cells which are believed to decline with age (Paratore et al., 2001), thus an apparent increase in Sox‐10 EGCs bodies in the adult samples.

In further analysis, we did not detect any sex‐related differences in the number of Sox‐10 IR EGCs in the plexus studied in contrast to what has been reported in the intermediate SMP from human ileum (Hoff et al., 2008). Although the present study reported no change in the overall number of Sox‐10 IR EGCs among the elderly, it does not necessarily eliminate the possibility that colonic function can be maintained as a result. Alterations in EGCs function as a result of influence of other factors such as inflammation or in disease condition could also significantly contribute to changes in colonic motility (see Grubišić et al., 2018).

We then assessed the density of S100‐IR EGCs per ganglionic area in the MP. This was to evaluate the distinct S100‐IR EGCs subpopulation within the ganglion (Type I) at the MP. The S100 marker identified both EGCs bodies and processes within the ganglia and the circular muscle. Therefore, a decline in density in the S100 IR in this present study attributes to a decrease in S100 protein expression in EGCs in the ROI investigated. Whether this subtype of S100‐IR EGCs in the ROI studied are vulnerable to age‐related decline with a concomitant functional change to the physiology of the colon, merits a further investigation. The use of densitometric analysis in assessing S100‐IR EGCs density in tissue section has been similarly performed in other studies (see Stenkamp‐Strahm et al., 2013). We believe the mechanism leading to a decline in density of S100‐IR EGCs among the elderly (but not Sox‐10 IR) requires further investigation.

In the myenteric ganglia of human proximal and distal colon, isolated by laser microdissection, quantitative PCR was unable to show a change in S100β mRNA with increasing age (Hetz et al., 2014), confirmed by Palmer et al. (Palmer et al., 2021) using the intact human colon. Nevertheless, analysis of S100‐IR EGCs per ganglionic area in the MP showed a statistically significant decline in density among the elderly. A decline in intraganglionic S100‐IR EGCs density has not previously been reported in aging human descending colon. Moreso, the loss of S100‐IR EGCs in the myenteric ganglia was consistent with studies using aged Fisher 344 rats (26‐month‐old vs 5–6‐month‐old) where a significant loss of enteric glia cell occurred in every region of the small and large intestine as determined by immunofluorescence (Phillips et al., 2004). By contrast, an increase in S100‐IR EGCs expression was found in the small and large intestine of the aged Thy1‐APP23 mouse (Van Ginneken et al., 2011); reasons for the difference are not understood.

To the best of our knowledge, our report is the first to analyze age‐related changes in the EGCs distribution within the musculature of human descending colon. In the adult (male and female) samples, there were more S100 IR EGCs located in the CM layer per unit area than in the *Taenia coli*, perhaps indicating a functional requirement for this regional EGCs distribution within the colonic wall. Another finding was that a loss of S100‐IR EGCs density was observed at the CM layer of the elderly compared to the adult group. Loss of EGCs in general has been equated to physiological change in the colon (e.g., motility; Bassotti et al., 2006; Bassotti et al., 2007). However, an important consideration in the present analysis with S100 antibodies is their low capacity to label all enteric glia in the colonic wall compared to the most extensive claims of completeness and specificity of Sox‐10 antibodies (Boesmans et al., 2015; Grundmann et al., 2019). In addition, S100 protein expression decreases with age therefore resulting in a lower identification of EGCs population than would otherwise occur in elderly samples (Chan et al., 2003).

A possible explanation for some discrepancies between the present study and previous studies may be found in the possibility that anti Sox‐10 and S100 may stain different populations of EGCs which are lost at different rates during aging. However, at present there is no evidence to support such a possibility. Another hypothesis is the existence of a compensatory maintenance in the number of Sox‐10 glial cell bodies within the MP of the elderly. It is not known whether the S100‐IR EGCs loss we see at the CM is due to apoptosis or another means, the mechanism of EGCs loss during aging at the muscularis region needs further investigation.

EGCs consists of an array of densely packed intermediate filaments capable of expressing the GFAP antibodies in humans and animals (Rothman et al., 1986). Based on paraffin embedded sections the present study reveals an absence of the expression of GFAP in the mucosa, submucosa, and the nerve plexuses except in the circular muscle layer where very few IR EGCs structures were identified. In contrast, GFAP expressing EGCs labeled the MP of the human esophagus (Nascimento et al., 2010), suggesting regional differences in the expression. Further, expression level of GFAP signal on human gut samples using immunofluorescence staining technique have been reported to be downregulated (Boesmans et al., 2015; Hoff et al., 2008), consistent to the findings in this present study. Our observation in the low expression of GFAP in our samples may support the notion of EGCs heterogeneity dynamically dictated by the unique microenvironment in the colonic wall (Boesmans et al., 2015). Regardless, GFAP is always present in the ENS in rodents (Chadi et al., 2004; Raab & Neuhuber, 2004; Stenkamp‐Strahm et al., 2013) and may show increased expression when the gut wall is compromised with inflammation or disease (von Boyen et al., 2011). In this context, caution is required when translating data from rodents to human study as differences in receptor expression (Nasser et al., 2006), channel expression (Costagliola et al., 2009) and physiology (Maudlej & Hanani, 1992) of distinct types of EGCs between species exists. In the analysis of colonic EGCs in healthy human adult bowel in a recent transcriptional profiling investigation, little to no GFAP‐IR were reported compared to that of S100 (Drokhlyansky et al., 2020; Kinchen et al., 2018), this finding in consistent with what has been reported in this present study may indicate that GFAP may not be a robust biomarker for the analysis of EGCs in human bowel at any age.

Although our current data suggest a decline in S‐100‐IR EGCs with age, the mechanism that contribute to myenteric glial cells loss is not well understood. In animal studies, dietary manipulation has been reported to influence EGCs numbers (Baudry et al., 2012; Stenkamp‐Strahm et al., 2013; van Haver et al., 2008). For example, in aging wistar rats (23 months old), caloric restriction (50% of normal diet) accentuated the loss of S100‐IR EGCs in the proximal colon compared to control group (Schoffen et al., 2014). Nevertheless, the present study reported a statistically insignificant decline of Sox‐10 IR EGCs numbers at both the MP and SMP.

The limitations of the current study include the relatively small numbers of human colonic tissues studied; a larger sample may be required to establish a more clinical relevance outcome. Nevertheless, power analysis from initial pilot studies provided the minimum samples needed to reach a biological significance. Moreover, due to morphological and discrete arrangement of ganglia particularly that of the submucous ganglia, the use of whole‐mount preparations (Wedel et al., 1999) may have been preferable. The issue of lack of pan‐EGCs biomarker is very important concern in the study of aging; for instance, the reliability of S100 and GFAP expression in human samples varies considerably compared to those in animal studies.

In conclusion, the present study has demonstrated the presence of Sox‐10‐, S100‐, and GFAP‐IR EGCs expression in human colon, which varies according to age and region of colon. Thus, there is a decline of S100‐IR EGCs density among the elderly in both the myenteric and CM which was not accompanied by a loss of Sox‐10‐IR EGCs in the MP. The lack of GFAP expression in the elderly samples may indicate that enteric glia cells are not activated with advanced age. Differences in the expression level of Sox‐10, S100 and GFAP support the notion of EGCs heterogeneities in the human colon.

## AUTHOR CONTRIBUTIONS

Nicholas Baidoo critically reviewed, designed and conducted the experiments, analyzed the data and cowrote the manuscript with Abi Belai and Gareth J Sanger, Abi Belai co‐designed the experiments, analyzed the data and supervised the overall project, Gareth J Sanger facilitated the identification, collection and governance of human tissue collection for this study, all authors participated in its construction and refinement.

## FUNDING INFORMATION

Self‐funded research project.

## CONFLICT OF INTEREST

On behalf of all authors, the corresponding author states that there is no conflict of interest.

## Supporting information


**Supplementary sheet 1** Human descending colonic tissues included in the study.

**Supplementary sheet 2** Identification of Sox‐10 immunoreactive (IR) structures within the submucosa of adult colon. Individual Sox‐10‐IR could be clearly identified by their intensely red stained cell bodies. Three different ganglia were identified within the submucosa. C1: Ganglia close to the muscularis mucosae (Mm), scale bar 20 μm; C2: those found in the middle (intermediate) of submucosa; scale bar 50 μm and C3: is the ganglia found close to inner circular muscle (CM); scale bar 50 μm. SMG: submucous ganglion.

## Data Availability

All data supporting the findings of this study are available from the corresponding author upon request.
