# Supplementary material for: Effect of old age on the subpopulations of enteric glial cells in human descending colon
Source: Glia. 2022 Sep 20;71(2):305–16. doi: 10.1002/glia.24272 (PMC10087700; doi:10.1002/glia.24272)
Supplement: Supplementary file 1 — Supplementary sheet 1 Human descending colonic tissues included in the study. [file GLIA-71-305-s001.docx]

**Supplementary sheet 1.**

Human descending colonic tissues included in the study.

| **Age (y)** | **Sex** | **Diagnosis** | **Comorbidity** | **Medication** |
| --- | --- | --- | --- | --- |
| 23 | F | Cancer | Unknown | Unknown |
| 39 | M | Cancer | Unknown | Cetirizine |
| 42 | F | Cancer | Unknown | Aspirin / Bisoprolol |
| 47 | F | Cancer | Unknown | Ramipril |
| 51 | M | Cancer | Unknown | Unknown |
| 52 | M | Cancer | Unknown | Unknown |
| 53 | F | Cancer | Sigmoid mass lesion | Unknown |
| 57 | M | Cancer | Adrenal adenoma | Ferrous fumarate |
| 59 | F | Cancer | Unknown | Unknown |
| 60 | M | Caner | Unknown | Unknown |
| 61 | F | Cancer | Disorder of peritoneum | Unknown |
| 61 | F | Cancer | Unknown | Atorvastatin |
| 63 | M | Cancer | Cholelithiasis | Statin |
| 66 | M | Cancer | Secondary lung cancer | Tramadol |
| 66 | F | Cancer | Unknown | Unknown |
| 70 | M | Cancer | Type 2 diabetes | Amitriptyline |
| 71 | M | Cancer | Peritoneal adhesion | Unknown |
| 72 | F | Cancer | Unknown | Unknown |
| 76 | M | Cancer | Unknown | Simvastatin |
| 77 | F | Cancer | Unknown | Unknown |
| 79 | M | Cancer | Unknown | Rivaroxaban |
| 80 | F | Cancer | Unknown | Unknown |
| 81 | M | Cancer | Fibroepithelial polyps | Apixaban |

*Unknown: No information on patient record at the time of consenting.
