# Supplementary material for: Effect of old age on the subpopulations of enteric glial cells in human descending colon
Source: Glia. 2022 Sep 20;71(2):305–16. doi: 10.1002/glia.24272 (PMC10087700; doi:10.1002/glia.24272)
Supplement: Supplementary file 2 — Supplementary sheet 2 Identification of Sox‐10 immunoreactive (IR) structures within the submucosa of adult colon. Individual Sox‐10‐IR could be clearly identified by their intensely red stained cell bodies. Three different ganglia were identified within the submucosa. C1: Ganglia close to the muscularis mucosae (Mm), scale bar 20 μm; C2: those found in the middle (intermediate) of submucosa; scale bar 50 μm and C3: is the ganglia found close to inner circular muscle (CM); scale bar 50 μm. SMG: submucous ganglion. [file GLIA-71-305-s002.docx]

**Supplementary sheet 2.0**


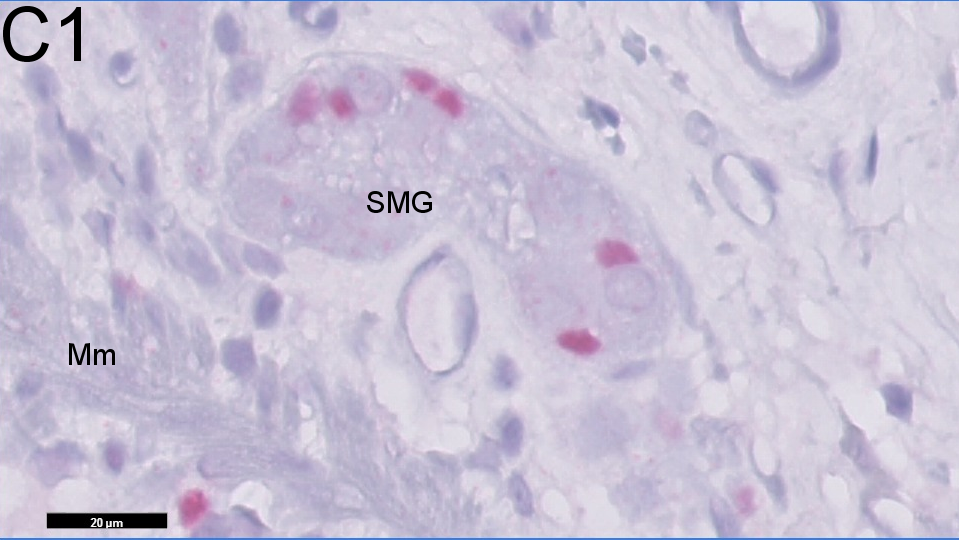


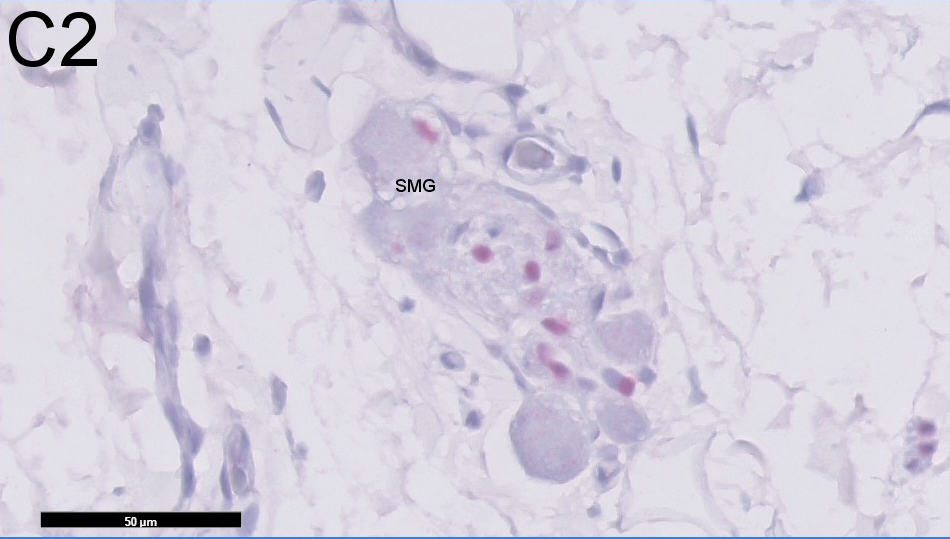


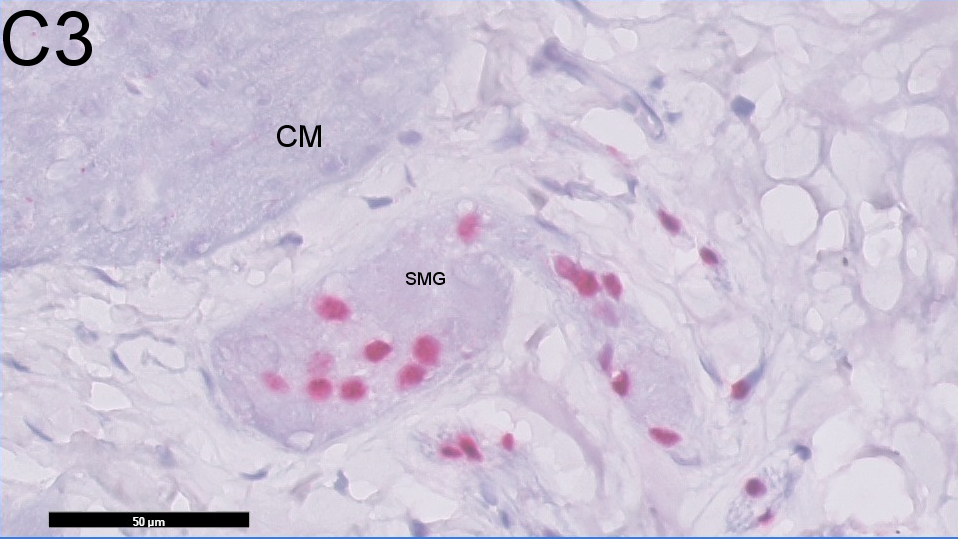


Identification of Sox-10 immunoreactive (IR) structures within the submucosa of adult colon. Individual Sox-10-IR could be clearly identified by their intensely red stained cell bodies. Three different ganglia were identified within the submucosa. **C1**: Ganglia close to the muscularis mucosae (Mm), scale bar 20 µm; **C2:** those found in the middle (intermediate) of submucosa; scale bar 50 µm and **C3**: is the ganglia found close to inner circular muscle (CM); scale bar 50 µm. SMG: submucous ganglion.
